# Hepatic Copper Accumulation Predicts Fibrosis Progression and Mortality in Patients with Metabolic Dysfunction-Associated Steatotic Liver Disease (MASLD)

**DOI:** 10.3390/nu17182923

**Published:** 2025-09-11

**Authors:** Suha Shabaneh, Elliot M. Berry, Ashraf Imam, Mohamad Suki, Ahmad Salhab, Abed Khalaileh, Rifaat Safadi

**Affiliations:** 1 Solid Organ Transplantation Unit, Department of General Surgery, Hadassah Medical Organization, Hadassah Hebrew University Medical Center, P.O. Box 12000, Jerusalem 91120, Israel; suhasuha1987@gmail.com (S.S.); ash_imam04@hotmail.com (A.I.); 2 Braun School of Public Health, The Hebrew University, Jerusalem 91904, Israel; elliotb@ekmd.huji.ac.il; 3 Liver Institution, Hadassah-Hebrew University Medical Center, Ein Karem, Jerusalem 91120, Israel; suki@hadassah.org.il (M.S.); ahmad.salhab@mail.huji.ac.il (A.S.); safadi@hadassah.org.il (R.S.)

**Keywords:** hepatic copper, MASLD, liver fibrosis, non-invasive fibrosis markers, nutrition, mortality, metabolic dysfunction

## Abstract

**Background:** Copper is an essential trace element involved in antioxidant defense and mitochondrial function. Evidence suggests that copper homeostasis may also influence metabolic liver diseases. We investigated the association between hepatic copper levels (HCLs) and liver-related outcomes among patients with Metabolic Dysfunction-Associated Steatotic Liver Disease (MASLD). **Methods:** In this retrospective cohort study, we analyzed 215 MASLD patients who underwent liver biopsy with copper quantification. Patients were categorized based on hepatic copper content; normal < 50 vs. high ≥ 50 μg/g dry tissue (165 vs. 50 patients, respectively). The primary outcomes were progression in non-invasive fibrosis score (FIB-4) and incidence of clinical events (cirrhosis, liver transplantation, cardiovascular events or death) during a median follow-up of 4.9 ± 4.2 years. Multivariable linear and logistic regression models were adjusted for metabolic and demographic confounders. **Results:** Both liver copper groups shared similar baseline characteristics. High hepatic copper levels independently predicted higher FIB-4 scores at the end of follow-up in the fully adjusted linear regression model (β = 0.41; 95% CI: 0.05–0.76; *p* = 0.026). Logistic regression confirmed that high HCLs were associated with significant FIB-4 deterioration (OR = 41.3; 95%; *p* = 0.008). Kaplan–Meier analysis revealed significantly reduced overall survival among patients with high HCLs (Log-Rank *p* = 0.034), and multivariable Cox regression showed a markedly increased mortality risk (HR = 18.51; 95%; *p* = 0.032). Subgroup analyses highlighted greater risk among females, patients with diabetes or dyslipidemia, and individuals of Arab ethnicity. **Conclusions:** Elevated hepatic copper levels are associated with long term worsened liver fibrosis and higher mortality in MASLD. These findings support hepatic copper as a potential nutritional biomarker for risk stratification. Further studies are needed to explore copper modulation as a therapeutic target in MASLD.

## 1. Introduction

Metabolic dysfunction-associated steatotic liver disease (MASLD), formerly classified as non-alcoholic fatty liver disease (NAFLD), has recently been redefined to better reflect its strong association with metabolic risk factors. MASLD now affects approximately 38% of adults globally, making it the most prevalent chronic liver disease worldwide [1]. This condition encompasses a broad spectrum of liver injury, ranging from simple steatosis to metabolic dysfunction-associated steatohepatitis (MASH), which can progress to advanced fibrosis, cirrhosis, and hepatocellular carcinoma [2]. Fibrosis stage has consistently emerged as the strongest predictor of liver-related and all-cause mortality in MASLD patients [3].

While traditional risk factors (such as obesity, type 2 diabetes mellitus, and dyslipidemia) are well-established contributors to disease progression, emerging data suggest that micronutrient status may also influence MASLD pathogenesis [4]. Copper, an essential trace element, plays a vital role in mitochondrial function, antioxidant defense (via superoxide dismutase), and connective tissue remodeling [5]. The liver is the central organ responsible for copper regulation, including uptake, storage, and biliary excretion [6]. Therefore, disturbances in copper homeostasis can have profound effects on hepatic structure and function.

Both copper deficiency and copper overload can induce hepatic injury, albeit through distinct mechanisms. Copper deficiency has been associated with impaired mitochondrial respiration, hepatic lipid accumulation, and steatosis [7,8], whereas copper overload promotes oxidative stress, inflammation, and fibrosis through reactive oxygen species generation and mitochondrial dysfunction [9,10]. Excessive hepatic copper accumulation is known to drive hepatotoxicity in Wilson’s disease, and recent studies suggest that subclinical copper imbalance may play a role in the natural history of MASLD [11,12].

Furthermore, a recently identified form of copper-induced cell death—known as cuproptosis—has been implicated in hepatic injury. Cuproptosis results from copper-dependent disruption of mitochondrial protein function and iron–sulfur cluster degradation, leading to non-apoptotic cell death in metabolically active tissues such as the liver [13].

Despite the biological plausibility, the relationship between hepatic copper and MASLD progression in humans remains poorly defined. Most studies have measured serum copper levels, which may not accurately reflect hepatic copper stores or bioactivity. Moreover, findings have been conflicting, with reports linking both copper deficiency and excess to MASLD severity [14].

In this study, we aimed to investigate the association between hepatic copper levels (measured directly from liver tissue) and liver fibrosis progression and clinical outcomes in patients with MASLD. We hypothesized that elevated hepatic copper would be independently associated with worsening fibrosis and increased mortality, thereby offering insight into copper as a nutritional and prognostic biomarker in metabolic liver disease.

## 2. Materials and Methods

### 2.1. Study Design and Population

We conducted a retrospective cohort study of adult patients (≥16 years) with biopsy-proven MASLD who underwent hepatic copper quantification at Hadassah Medical Center between January 2009 and December 2023. Inclusion criteria were: (1) histological confirmation of MASLD (steatosis ≥5% of hepatocytes with ≥1 cardiometabolic risk factor); (2) available hepatic copper level (HCL) measurement; and (3) complete baseline clinical, laboratory, and follow-up data. Exclusion criteria included other chronic liver diseases (e.g., viral hepatitis, autoimmune hepatitis, hemochromatosis, alcoholic liver disease), hepatic copper > 250 µg/g dry tissue (suggestive of Wilson’s disease), liver malignancy at baseline, or insufficient data.

Wilson’s disease was excluded in all cases according to the EASL Clinical Practice Guidelines [15]. Patients with hepatic copper levels > 250 μg/g dry weight, or those with clinical suspicion of Wilson’s disease, were excluded. In patients with levels < 250 μg/g, the absence of supportive clinical findings (neurological symptoms, Kayser–Fleischer rings, hemolytic anemia, low ceruloplasmin) allowed safe exclusion, as recommended by the guidelines.

### 2.2. Liver Biopsy and Histological Assessment

Percutaneous liver biopsies were performed under ultrasound guidance using a 16-gauge needle. Biopsy specimens were fixed in formalin, embedded in paraffin, and stained with hematoxylin-eosin and Masson’s trichrome. Histological evaluation was conducted by experienced hepato-pathologists blinded to clinical data, assessing steatosis, lobular inflammation, hepatocellular ballooning, and fibrosis stage according to the NAFLD Activity Score (NAS) and the Kleiner classification system [16].

### 2.3. Hepatic Copper Measurement

Hepatic copper concentration was measured using flame atomic absorption spectroscopy on dried liver tissue samples. The entire core of the liver biopsy was utilized to minimize sampling variability, as recommended in previous studies. Tissue samples were dried at 65 °C until a constant weight was achieved, and copper content was expressed in micrograms per gram (μg/g) of dry weight. Quality control measures included the use of certified reference materials and duplicate analyses to ensure accuracy and precision. Results are expressed in micrograms of copper per gram of dry liver tissue (µg/g). Based on literature and local reference ranges [17], patients were dichotomized into “normal” HCL (<50 µg/g) and “high” HCL (≥50 µg/g). Values over 250 µg/g of dry tissue were excluded from the database as indicative of Wilson’s disease. The method followed standard reference protocols (PerkinElmer, Waltham, MA, USA, AAS with flame atomizer), with intra- and inter-assay CVs < 5%. Detection limit was 1 µg/g dry tissue.

### 2.4. Clinical and Laboratory Data Collection

Demographic and clinical data, including age, sex, ethnicity, body mass index (BMI), smoking history, presence of diabetes mellitus, hypertension, and dyslipidemia, were extracted from electronic medical records. Laboratory parameters collected at baseline and during follow-up included liver function tests, fasting glucose, lipid profile, and platelet count. The Fibrosis-4 (FIB-4) score is a non-invasive tool used to estimate the presence of advanced liver fibrosis. It is calculated using a formula that considers the patient’s age, blood tests for AST (Aspartate Aminotransferase), ALT (Alanine Aminotransferase), and platelet count. The FIB-4 score helps determine if further evaluation or treatment for liver disease is needed, especially in cases of MASLD. Thus, the FIB-4 index was calculated at biopsy time point and at end of follow up.

### 2.5. Outcome Definitions

- Fibrosis progression: Primary outcome assessed by change in non-invasive FIB-4 score between baseline and end of follow-up (EoFU); deterioration defined as EoFU FIB-4 > baseline FIB-4.
- Clinical events: Secondary outcomes included time to all-cause mortality, liver transplantation, new cirrhosis, and cardiovascular events. Patients were censored at last clinic visit if no event occurred.

### 2.6. Statistical Analysis

Continuous variables are presented as mean ± standard deviation (SD) or median (interquartile range; IQR) and categorical variables as counts and percentages. Between-group comparisons used Student’s *t*-test or Mann–Whitney U-test for continuous data and chi-square test for categorical data.

- Linear regression: Multivariate linear regression analyses were conducted to identify independent predictors of fibrosis progression, adjusting for potential confounders. EoFU FIB-4 was modeled as a function of HCL category (high vs. normal), and adjusting sequentially for baseline FIB-4, age, sex, ethnicity, smoking status, BMI, hypertension, diabetes/impaired fasting glucose, dyslipidemia, and follow-up duration.
- Logistic regression: Estimated odds ratios (OR) for FIB-4 deterioration with the same covariate adjustment.
- Survival analysis: Kaplan–Meier curves and log-rank tests compared event-free survival by HCL group. Cox proportional hazards models estimated hazard ratios (HR) for time-to-event outcomes and mortality, adjusting for potential confounders. Proportionality assumptions were checked using Schoenfeld residuals.

Missing data were handled by listwise deletion. All tests were two-sided, with *p* < 0.05 considered significant. Analyses were performed using SPSS v.23 (IBM, Armonk, NY, USA).

### 2.7. Ethical Approval

The study protocol was approved by the Hadassah Medical Center Institutional Review Board (Approval No. 036218 on 13 March 2019). Due to the retrospective nature and use of de-identified data, informed consent was waived.

## 3. Results

### 3.1. Participant Baseline Characteristics

A total of 215 MASLD patients were included (Table 1), with a mean age of 40.0 ± 14.3 years (ranging from 16 to 73.5 years), and 60% of the patients were male and 66% were Jewish. The mean BMI was 29.6 ± 4.7 kg/m^2^, and 22% were current or former smokers. Median follow-up was 4.88 ± 4.15 years. Hepatic copper levels were <50 µg/g dry tissue in 165 patients (76.7%) and ≥50 µg/g in 50 patients (23.3%). Baseline demographics, metabolic comorbidities (hypertension, diabetes/impaired fasting glucose, dyslipidemia), and FIB-4 scores did not differ significantly between the normal- and high-copper groups (all *p* > 0.05). All 215 cases had EoFU survival outcomes; 132 cases (61.4%) had baseline FIB-4 and 127 (59.1%) cases also had EoFU FIB-4. A breakdown of absolute copper levels and associated baseline histological and clinical data for each patient is provided in Supplementary Figure S1.

### 3.2. Hepatic Copper and Fibrosis Progression

A total of 127 patients had baseline and EoFU FIB-4 with an average follow-up time of 5.1 ± 4.3 years. In univariable analysis (Table 2a), HCLs ≥ 50 µg/g was strongly associated with higher FIB-4 at end of follow-up (β = 0.75; 95% CI: 0.34–1.16; *p* < 0.001). Similarly, other factors predicted EoFU FIB-4 (baseline FIB-4, age, ethnicity and hypertension), while others did not (sex, smoking, BMI, diabetes/impaired fasting glucose, dyslipidemia and follow-up duration).

To predict EoFU FIB-4, four linear regression models used (Table 2b) according to relevant baseline variables (including baseline FIB-4, age, sex, ethnicity, smoking, BMI, hypertension, diabetes/impaired fasting glucose, dyslipidemia, and follow-up duration). Model-1 included only HCLs subgroups as the predictor variable. In Model-2, baseline FIB-4 was added. In Model-3, baseline age, gender, ethnicity, smoking and co-morbidities (diabetes/impaired fasting glucose, hypertension, and dyslipidemia) added. Due to the low numbers, BMI was included in a separate step in Model-4. Although all models achieved the same significant prediction outcome for HCLs subgroups, Model-4 kept the robust analysis. Accordingly, in a fully adjusted linear regression models, high HCLs remained an independent predictor of increased EoFU FIB-4: Model 1 (HCLs only): β = 0.75 (95% CI: 0.34–1.16; *p* < 0.001), Model 2 (+baseline FIB4): β = 0.57 (95% CI: 0.17–0.96; *p* = 0.005), Model 3 (+age, gender, ethnicity, smoking, metabolic factors): β = 0.51; *p* = 0.016 and Model 4 (+BMI): β = 0.41 (95% CI: 0.05–0.76; *p* = 0.026). Model-4 explained 76.3% of variance (adjusted R^2^ = 0.763), demonstrating a strong independent association between high hepatic copper and fibrosis progression.

To predict the EoFU FIB-4 deterioration, HCLs and odds of fibrosis deterioration analyzed using Logistic Regression models. Model-1 to Model-4 followed the same orders used in the linear regression analysis. Logistic regression models (Table 2c) similarly demonstrated that patients with HCLs ≥ 50 µg/g had dramatically higher odds of FIB-4 deterioration (EoFU FIB-4 > baseline FIB-4) in the fully adjusted models. It includes Model-1 (HCLs only): OR = 13.09; *p* = 0.017), but namely Model-4 (OR = 41.3; *p* = 0.008). The baseline FIB4 was not significant in any model. The diabetes/impaired fasting glucose only approached a protective trend (OR = 0.17; *p* = 0.061).

**Figure 1:**
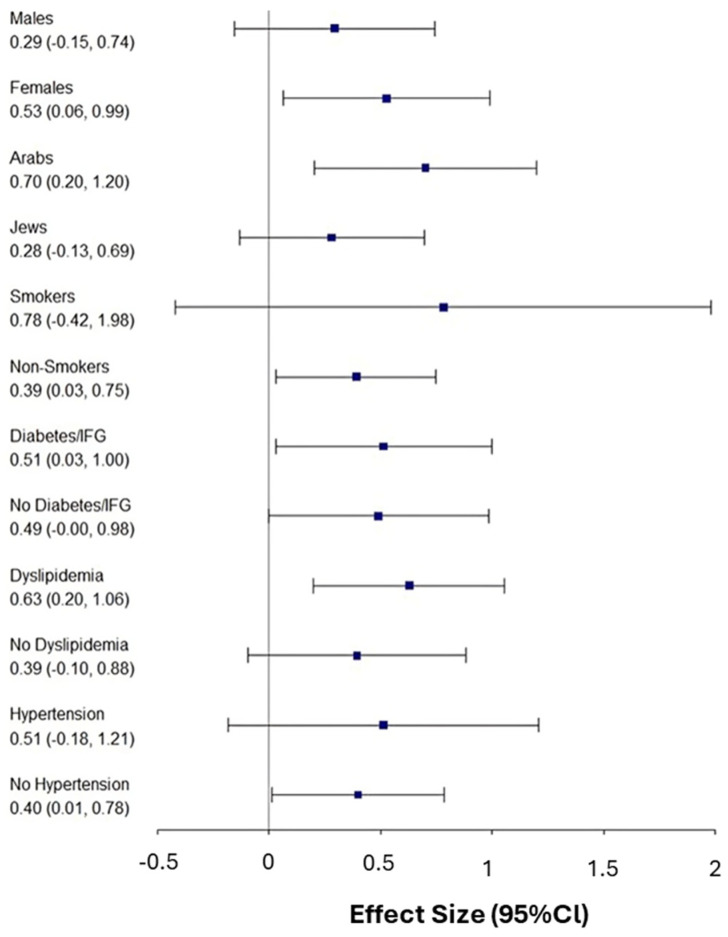
The forest plot illustrations of subgroups. The results from the subgroup analysis of the logarithm of FIB-4 score by two groups categorized by HCLs (≥50 vs. <50) reveal varying effect sizes across different demographic and clinical subgroups. The forest plot visualization illustrates that certain subgroups significantly strengthen the positive association between FIB-4 progression and elevated HCLs.

A forest plot analysis was conducted to explore subgroup findings, whether specific subgroups exhibited stronger associations between high HCLs subgroup and FIB-4 score progression at the end of follow-up (Figure 1). The visualization revealed that certain demographic and metabolic subgroups significantly enhanced the positive relationship between elevated HCLs and liver fibrosis progression. Specifically, the association was significantly stronger among females (β = 0.53; 95% CI: 0.06–0.99), Arab participants (β = 0.70; 95% CI: 0.20–1.20), individuals with diabetes or impaired fasting glucose (β = 0.51; 95% CI: 0.03–1.00), and those with dyslipidemia (β = 0.63; 95% CI: 0.20–1.06). These findings suggest that elevated hepatic copper may be particularly detrimental in these subpopulations, amplifying the risk of fibrosis progression. Although smokers demonstrated a relatively large effect size (β = 0.78), the confidence interval was wide and crossed zero, indicating a lack of statistical significance. Conversely, other subgroups—such as males, Jewish participants, non-smokers, individuals without hypertension, and those with normal glucose and lipid homeostasis—exhibited effect sizes closer to zero, implying a weaker or non-significant relationship between HCLs and FIB-4 progression in these populations.

### 3.3. Clinical Events and Survival Analysis

**Figure 2:**
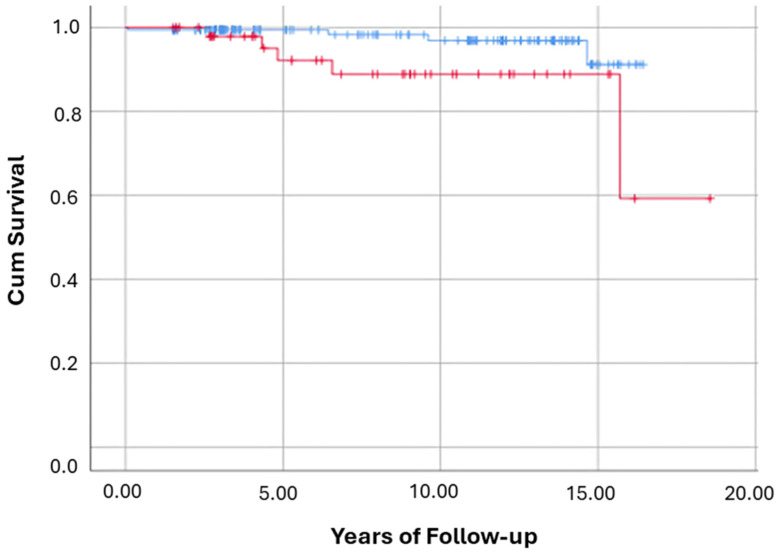
Kaplan–Meier curves for all-cause mortality by hepatic copper group. This Kaplan–Meier survival curve illustrates the cumulative survival probability over time for participants with hepatic copper < 50 µg/g (blue line) vs. ≥50 µg/g (red line). X-axis = years of follow-up; Y-axis = cumulative survival probability. Log-Rank test: *p* = 0.034.

All-Cause mortality and hepatic copper evaluated in 212 participants. Over follow-up, 9 patients died (4.2%): 4/162 (2.5%) in the normal-copper group versus 5/50 (10%) in the high-copper group. Kaplan–Meier analysis (Figure 2) showed significantly worse overall survival in the high-copper group (Log-Rank *p* = 0.034). In multivariable Cox regression adjusting for demographic and clinical covariates (Model-3), HCLs ≥ 50 µg/g was associated with an 18.5-fold increased hazard of all-cause mortality (HR = 18.51; *p* = 0.032). There were no significant differences between copper groups in the incidence of new cirrhosis (Log-Rank *p* = 0.734) or cardiovascular events (Log-Rank *p* = 0.747) during follow-up.

We constructed a series of Cox proportional hazards models to evaluate predictors of all-cause mortality (Table 3). Model 1 included only HCLs category: HR = 3.78; *p* = 0.048. Model 2 added demographic factors (age, sex, ethnicity, smoking): HCLs retained a strong effect (HR ≈ 16–18); age also emerged as significant (HR = 1.16 per year; *p* = 0.005), and smoking status predicted higher risk (HR = 22.6; *p* = 0.011). Model 3 further adjusted for comorbidities (diabetes, dyslipidemia, hypertension) and baseline fibrosis stage: HCLs ≥ 50 µg/g remained independently associated with mortality (HR = 18.51; *p* = 0.032), while the effects of age and smoking were attenuated but still significant for smoking (HR = 16.61; *p* = 0.048). The Nagelkerke R^2^ for Model 3 was 0.593, indicating a robust model fit. Sex, ethnicity, metabolic comorbidities, and baseline fibrosis stage did not reach statistical significance in the fully adjusted model, suggesting that hepatic copper and smoking were the primary independent determinants of mortality in this cohort.

**Figure 3:**
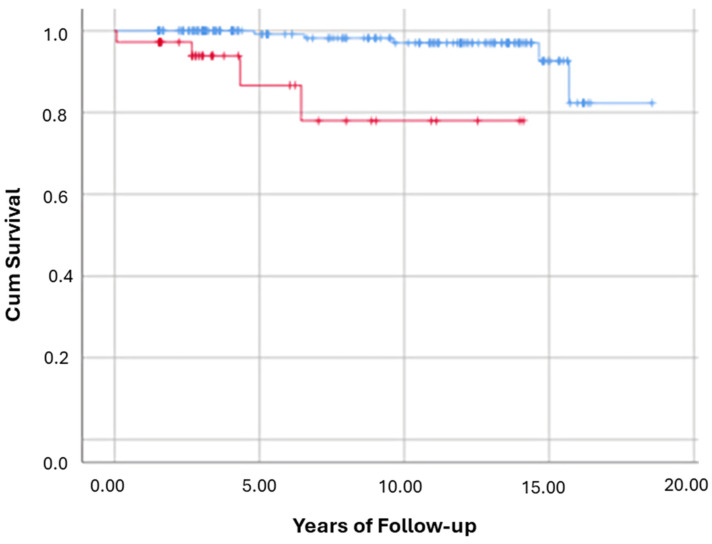
Kaplan–Meier for the risk of all-cause mortality among participants with Fibrosis stages (0 to 2) vs. Fibrosis stages (3 to 4). This Kaplan–Meier survival curve illustrates the cumulative survival probability over time for participants with Fibrosis stages 0 to 2 (blue line) vs. Fibrosis stages 3 to 4 (red line). X-axis = years of follow-up; Y-axis = cumulative survival probability. Log-Rank test: *p* = 0.00.

Fibrosis-Stage stratified mortality: Out of the 212 participants in the current cohort, five of 176 with low baseline histological fibrosis stages 0 to 2 died (2.8%) at the EoFU. While 4/36 participants with advanced fibrosis stages 3 or 4 died (11.1%) at the EoFU. The Log Rank test result (*p* < 0.01) indicates that the survival distributions for the two groups (low fibrosis stages) vs. (advanced fibrosis stages) are significantly different (Figure 3). Results of Cox regression suggests that being in advanced Fibrosis stage according to biopsy increases the crude hazard to all-cause mortality by 12.2 (95% CI: 2.655–56.02, *p* = 0.001).

Participants were then analyzed in different sub-groups according to baseline histologic Fibrosis stages, low fibrosis stages 0 and 1, significant Fibrosis stage 2, and participants with advanced Fibrosis stages 3 and 4. In F2 cases, mortality rate was 40% (2/5) with high HCLs vs. 2.6% (1/38) with normal HCLs (*p* = 0.003). However, no statistically significant mortality differences observed within F3–F4 (3/35; 8.6% vs. 2/141; 1.4%, *p* = 0.151) and F0–F1 Fibrosis (1/30; 3% vs. 1/102; 1%, *p* = 0.842).

**Figure 4:**
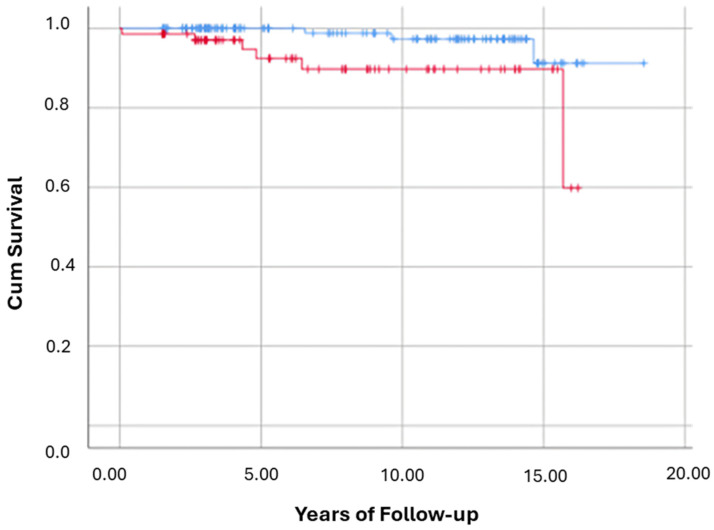
Kaplan–Meier Curves for All-Cause Mortality by Ethnicity. This Kaplan–Meier survival curve illustrates the cumulative survival probability for all-cause mortality over time from diagnosis to the end of follow-up, stratified by Arab and Jewish ethnicities. Cumulative survival for Jewish (blue line) vs. Arab (red line) participants. Log-Rank test: *p* = 0.018.

Ethnicity and mortality: Among the 212 participants included in the survival analysis, 3 out of 141 Jewish participants (2.1%) and 6 out of 71 Arab participants (8.5%) died by the end of follow-up (EoFU). Kaplan–Meier survival curves demonstrated a significantly lower survival probability in the Arab group compared to the Jewish group (Log-Rank test: *p* = 0.018; Figure 4), indicating a statistically significant difference in survival distributions between the two ethnic groups. Cox proportional hazards regression further supported these findings. In unadjusted analysis, Arab ethnicity was associated with a significantly increased risk of all-cause mortality, with a crude hazard ratio (HR) of 4.60 (95% CI: 1.15–18.50; *p* = 0.031). This suggests that participants of Arab ethnicity had more than a four-fold higher risk of mortality compared to their Jewish counterparts during the follow-up period.

**Figure 5:**
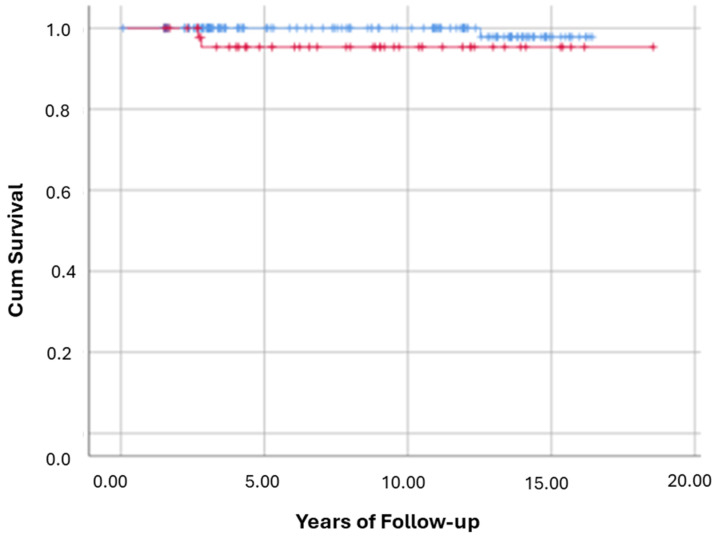
Kaplan–Meier Curves for Liver Transplantation by HCLs: This Kaplan–Meier survival curve illustrates the cumulative survival probability over time from diagnosis to the end of follow-up, stratified by normal and high HCLs. Event-free survival for transplantation in participants with hepatic copper <50 µg/g (blue line) vs. ≥50 µg/g (red line). Log-Rank test: *p* = 0.052.

Liver Transplantation: Among the 212 participants included in the time-to-event analysis, three individuals underwent liver transplantation during follow-up. Two of these occurred in the high-HCLs group (≥50 µg/g), and one in the normal-HCLs group (<50 µg/g). Although the total number of events was small, the Kaplan–Meier curves suggest a trend toward earlier transplantation in the high-copper cohort (Log-Rank *p* = 0.052; Figure 5), indicating that elevated hepatic copper may be associated with accelerated progression to end-stage liver disease requiring transplantation.

**Figure 6:**
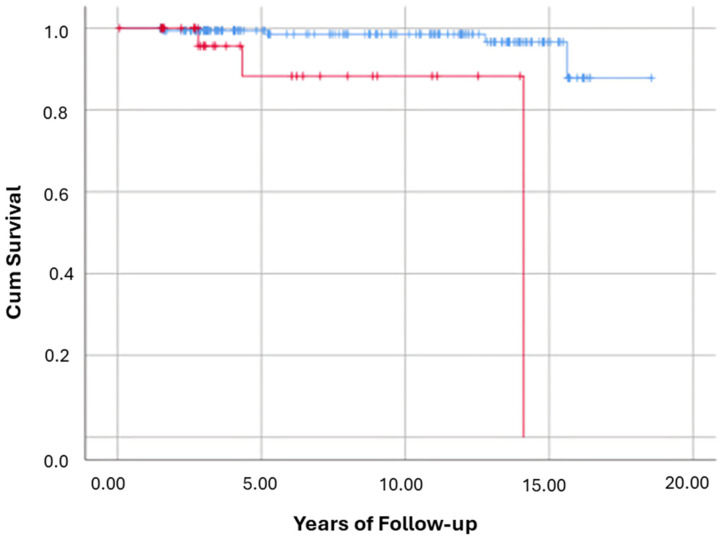
Kaplan–Meier Curves for Cardiovascular Events by Baseline Fibrosis Stage (Fibrosis stages 0 to 2 vs. Fibrosis stages 3 to 4): This Kaplan–Meier survival curve illustrates the cumulative survival probability of cardiovascular event outcome over time from diagnosis to the end of follow-up, stratified by high and low Fibrosis stages. Cumulative incidence of cardiovascular events in participants with low fibrosis (F0–F2; blue line) vs. advanced fibrosis (F3–F4; red line). Log-Rank test: *p* < 0.001.

Cirrhosis and Cardiovascular Events: We next examined the incidence of new cirrhosis and cardiovascular events by HCL group. Among 28 high-HCLs patients, one (3.6%) developed cirrhosis during follow-up versus one of 98 normal-HCLs patients (1.0%), with no statistically significant difference in cirrhosis-free survival (Log-Rank *p* = 0.734). Similarly, cardiovascular event rates did not differ meaningfully between groups (Log-Rank *p* = 0.747), suggesting that hepatic copper accumulation primarily impacts fibrotic progression rather than de novo cirrhosis or macrovascular complications in this cohort. To assess whether histologic fibrosis stage predicted cardiovascular risk, we stratified participants by baseline biopsy stage. Among 176 patients with low fibrosis (F0–F2), four (2.3%) experienced cardiovascular events, whereas three of 35 patients with advanced fibrosis (F3–F4; 8.6%) did so. The difference in event-free survival was significant (Log-Rank *p* < 0.001; Figure 6). In unadjusted Cox analysis, advanced fibrosis conferred an 11.34-fold increased hazard of cardiovascular events (HR = 11.34; *p* = 0.005), underscoring fibrosis stage as a key driver of vascular risk in MASLD.

For clarity, Figure 2, Figure 3, Figure 4, Figure 5 and Figure 6 represent complementary but separate analyses.

## 4. Discussion

In this retrospective cohort of 215 patients with biopsy-confirmed metabolic dysfunction-associated steatotic liver disease (MASLD), we demonstrate that elevated hepatic copper levels (HCLs ≥ 50 µg/g dry tissue) are independently associated with accelerated fibrosis progression and markedly worse clinical outcomes, including a dramatic increase in all-cause mortality and a trend toward earlier transplantation. These findings underscore the potential role of hepatic copper as both a prognostic biomarker and a therapeutic target in MASLD.

Our cohort analysis demonstrates that elevated hepatic copper levels (HCLs ≥ 50 µg/g) are strongly associated with liver fibrosis progression and adverse outcomes in MASLD. Even after adjusting for baseline fibrosis, demographics, metabolic factors, and BMI, high HCLs remained an independent predictor of higher FIB-4 scores at follow-up. This association was especially pronounced in subgroups such as females, Arabs, and individuals with diabetes or dyslipidemia, suggesting that copper’s fibrogenic effect may be modulated by biological and metabolic context. Logistic models further supported this relationship, with high HCLs significantly increasing the odds of fibrosis worsening over time. These data align with rodent studies show that copper overload drives hepatic stellate cell activation and collagen deposition via mitochondrial dysfunction and oxidative stress [18,19].

High HCLs also predicted poorer survival; patients with elevated copper had significantly higher mortality and a trend toward earlier liver transplantation. Although the numbers were small, these findings suggest that copper overload may accelerate disease progression. However, the incidence of new cirrhosis or cardiovascular events did not differ by copper level, highlighting that hepatic copper is more closely tied to fibrosis and survival than macrovascular complications. In contrast, advanced fibrosis itself strongly predicted cardiovascular events, underscoring the importance of fibrosis staging in broader risk assessment. Hepatic copper provides prognostic information beyond established noninvasive markers [20].

There are ample biological mechanisms that may explain our findings. Copper functions as a cofactor for cytochrome c oxidase and superoxide dismutase [21]. Excess intrahepatic copper may catalyze Fenton-type reactions, producing reactive oxygen species that trigger hepatocyte injury and stellate cell activation. The recently described “cuproptosis” pathway—copper-induced aggregation of mitochondrial lipoylated proteins—may further contribute to hepatocyte death and fibrogenesis [22].

The current cohort has strengths and limitations. Key strengths include direct hepatic copper measurement, histological MASLD confirmation, comprehensive multivariable adjustment, and long follow-up. Limitations are its retrospective, single-center design, modest event counts for transplantation and cirrhosis, missing BMI in some models, and lack of dietary copper intake data. Although comprehensive diagnostic workup for Wilson’s disease (including biochemical, hematological, ophthalmological, urinary copper exception and genetic assessment when indicated) was performed according to EASL guidelines, the possibility of subclinical Wilson’s disease cannot be completely excluded. This limitation, together with the retrospective and single-center design, may reduce the generalizability of our findings. We acknowledge that dichotomizing hepatic copper at 50 µg/g may oversimplify a continuum. Nonetheless, sensitivity analyses using copper as a continuous variable confirmed the same direction of association. Thus, the observed effects are unlikely to be driven by arbitrary cutoff clustering. Furthermore, we did not assess hepatic iron or zinc in this cohort. Future studies should evaluate whether combined profiling of multiple metals could provide stronger predictive value than copper alone. However, the results show clinical implications and future directions. Our findings support incorporating hepatic copper assessment into MASLD risk stratification, especially for patients with moderate fibrosis, women, ethnic minorities, and those with metabolic comorbidities. Therapeutic strategies to modulate copper homeostasis—dietary counseling, zinc supplementation, or chelation—should be explored in prospective clinical trials. Further mechanistic research is required to validate copper’s role in fibrogenesis and to develop targeted interventions.

## 5. Conclusions

In this retrospective cohort of patients with biopsy-proven MASLD, elevated hepatic copper levels (≥50 µg/g dry tissue) emerged as an independent predictor of fibrosis progression and significantly higher all-cause mortality, even after adjusting for demographic and metabolic risk factors. These findings highlight hepatic copper as a potential nutritional biomarker with prognostic value in MASLD, particularly in high-risk subgroups such as women, patients with diabetes or dyslipidemia, and those of Arab ethnicity. Incorporating hepatic copper assessment into clinical evaluation may improve risk stratification beyond conventional fibrosis markers. Future prospective and interventional studies are warranted to clarify the mechanistic role of copper in fibrogenesis and to explore therapeutic strategies aimed at modulating copper homeostasis as a means to mitigate disease progression and improve survival outcomes in MASLD.

## Figures and Tables

**Table 1:** Baseline characteristics of HCLs subgroups (<50 vs. ≥50 µg/g of dry tissue).

	HCLs <50 µg/g N = 165	HCLs ≥50 µg/g N = 50	Total N = 215	*p* Value
Age, years (mean ± SD)	40.6 ± 14.4	38.01 ± 13.72	40.02 ± 14.30	0.258
Male sex, n (%)	95 (57.6%)	34 (68%)	129 (60%)	0.241
Ethnicity: Jewish, n (%)	111 (67.3%)	31 (62%)	142 (66%)	0.5
Ethnicity: Arab, n (%)	54 (32.7%)	19 (38%)	73 (34%)	
Smoking, n (%)	25 (23.4%) N = 107	6 (19.4%) N = 31	31 (22.5%) N = 138	0.808
BMI, kg/m^2^ (mean ± SD)	29.1 ± 4.85 N = 77	31.04 ± 3.7 N = 16	29.6 ± 4.7 N = 93	0.141
FIB-4 (Mean ± SD)	1.90 ± 0.189 N = 132	1.93 ± 0.22 N = 36	1.92 ± 0.22 N = 168	0.286
Hypertension, n (%)	35 (27.3%) N = 128	7 (22.6%) N = 31	42 (26.4%) N = 159	0.657
Diabetes/IFG, n (%)	53 (34.9%) N = 152	11 (24.4%) N = 45	64 (32.5%) N = 197	0.210
Dyslipidemia, n (%)	72 (63.2%) N = 114	14 (50%) N = 28	86 (60.6%) N = 142	0.282
Follow-up time (mean ± SD), years	5.06 ± 4.3 N = 114	4.26 ± 3.49 N = 32	4.88 ± 4.15 N = 146	0.286

Chi-square tests and independent *t*-tests used to compare gender, smoking status, race, dyslipidemia, hypertension, diabetes mellitus, body mass index (BMI), baseline age and FIB-4 values and follow-up time range. Results are shown as N = number of individuals; Mean ± SD = Average ± Standard Deviation; % = Percentage and *p* values indicating statistical significance.

**Table 2:** (**a**) Univariate Analysis for predicting FIB-4 at end of follow-up. (**b**) Multivariable Linear Regression Models Predicting Fibrosis Progression. Effect of Hepatic Copper Levels and covariates on FIB-4 at End of Follow-Up. (**c**) Multivariable Logistic Regression for fibrosis deterioration. Odds of FIB-4 Deterioration by Hepatic Copper and clinical covariates.

(**a**)								
			**Beta**	**95% CI ^1^**		***p* Value**		
HCLs > vs. <50 mcg/g			0.75	0.34, 1.16		**<0.001**		
Baseline FIB-4			0.85	0.74, 0.97		**<0.001**		
Age			0.03	0.02, 0.05		**<0.001**		
Gender			0.01	−0.34, 0.36		0.959		
Ethnicity (Arab vs. Jew)			0.36	0.01, 0.71		**0.044**		
Smoking status			0.42	−0.13, 0.96		0.141		
Diabetes/Impaired Fasting Glucose			0.36	−0.01, 0.73		0.056		
Dyslipidemia			0.40	−0.06, 0.87		0.093		
Hypertension			0.74	0.33, 1.14		**0.001**		
Body Mass Index			−0.02	−0.07, 0.02		0.357		
^1^ CI = Confidence Interval The Outcome variable: natural logarithm of FIB-4 at EoFU.								
(**b**)								
	**Model 1**				**Model 2**			
**Predictors**	**Estimates**	**CI**		***p***	**Estimates**	**CI**		***p***
(Intercept)	1.10	−0.09–0.29		0.316	0.08	−0.04–0.19		0.207
Copper Category ≥ 50 vs. <50 mcg\g	0.75	0.34–1.16		**<0.001**	0.37	0.11–0.63		**0.006**
BaselineFib4FU					0.82	0.71–0.93		**<0.001**
Age								
Gender (ref = female)								
Ethnicity (ref = Jews)								
Smoking								
DM/IFG = 1								
Dyslipidemia								
HTN								
Follow up time, years								
BMI								
Observations	127				124			
R^2^/R^2^ adjust	0.094/0.087				0.661/0.655			
	**Model 3**				**Model 4**			
**Predictors**	**Estimates**	**CI**		***p***	**Estimates**	**CI**		***p***
(Intercept)	−0.03	−0.82–0.77		0.949	0.21	−1.30–1.73		0.779
Copper Category ≥ 50 vs. <50 mcg\g	0.49	0.16–0.82		**0.004**	0.41	0.05–0.76		**0.026**
BaselineFib4FU	0.98	0.80–1.16		**<0.001**	1.03	0.84–1.22		**<0.001**
Age	−0.01	−0.02–0.01		0.372	−0.01	−0.02–0.01		0.490
Gender (ref = female)	0.01	−0.28–0.29		0.962	0.07	−0.24–0.38		0.670
Ethnicity (ref = Jews)	0.25	−0.03–0.53		0.084	0.15	−0.15–0.44		0.316
Smoking	0.30	−0.04–0.64		0.082	0.35	−0.00–0.71		0.052
DM/IFG = 1	−0.35	−0.669–−0.01		**0.043**	−0.19	−0.57–0.19		0.325
Dyslipidemia	0.23	−0.10–0.56		0.162	0.23	−0.18–0.64		0.263
HTN	0.31	0.03–0.66		0.072	0.12	−0.26–0.51		0.517
Follow up time, years	0.02	−0.02–0.06		0.327	0.01	−0.04–0.05		0.687
Observations	81				59			
R^2^/R^2^ adjust	0.760/0.725				0.804/0.763			
(**c**)								
	**Fib-4 BL/FU > 1 IS 1**		**Fib-4 BL/FU > 1 IS 1**		**Fib-4 BL/FU > 1 IS 1**		**Fib-4 BL/FU > 1 IS 1**	
**Predictors**	**OR**	***p***	**OR**	***p***	**OR**	***p***	**OR**	***p***
(Intercept)	1.11	0.612	1.12	0.591	0.15	0.252	4.22	0.684
Copper Category ≥ 50 vs. <50 mcg\g	13.09	0.017	15.16	**0.013**	33.37	**0.013**	41.3	**0.008**
BaselineFib4FU			0.45	**0.001**	0.60	0.185	0.63	0.353
Age					1.00	0.970	0.99	0.76
Gender (ref = female)					2.36	0.212	1.81	0.423
Ethnicity (ref = Jews)					2.07	0.235	1.86	0.40
Smoking					3.19	0.163	3.13	0.182
DM/IFG = 1					0.20	**0.066**	0.17	0.061
Dyslipidemia					3.81	0.248	4.42	0.22
HTN					3.36	0.22	4.64	0.122
Follow up time, years					1.13	0.273	1.11	0.362
BMI							0.89	0.15
Observations	124		124		81		59	
R^2^	0.203		0.242		0.442		0.476	

(**a**) shows the univariate analysis results evaluating the association between various predictors and FIB-4 scores at the end of the follow-up period (calculated as Ln-FIB-4). The table provides the beta coefficients (β), 95% confidence intervals (CI), and *p*-values for each variable. A positive beta value indicates an increase in FIB-4 with higher levels of the predictor, while a negative beta indicates a decrease. (**b**) represents results of four successive linear regression models estimating the association between high hepatic copper (HCLs ≥ 50 vs. <50 µg/g) and the natural log of FIB-4 at end-of-follow-up. Model 1 includes only HCLs; Model 2 adds baseline FIB-4; Model 3 further adjusts for age, sex, ethnicity, smoking, diabetes/impaired fasting glucose, dyslipidemia, hypertension, and follow-up time; Model 4 adds body mass index. Displayed are regression coefficients (β), 95% confidence intervals (CI), *p*-values, R^2^ and adjusted R^2^. (**c**) presents the logistic regression models predicting the binary outcome EoFU FIB-4 > baseline FIB-4. Model 1 includes only HCLs; Model 2 adds baseline FIB-4; Model 3 adds demographics and metabolic comorbidities; Model 4 adds body mass index. Odds ratios (OR), 95% CIs, and *p*-values are shown for each predictor. Statistically significant predictors are bolded.

**Table 3:** Cox Regression Models for All-Cause Mortality.

	Model 1		Model 2		Model 3	
Predictors	HR	*p*	HR	*p*	HR	*p*
HCLs ≥ vs. < than 50 µg/g	3.78	**0.048**	10.09	**0.029**	18.51	**0.032**
Gender (ref = female)			0.98	0.988	2.21	0.625
Ethnicity (ref = Jew)			7.91	0.072	9.99	0.144
Smoking Status			22.59	**0.011**	16.61	**0.048**
Age at Biopsy			1.16	**0.005**	1.12	0.099
Diabetes Mellitus or Impaired Fasting Glucose					0.31	0.328
Dyslipidemia					10.19	0.135
Hypertension					4.14	0.230
Fibrosis stage 3 or 4 by Biopsy						0.625
Observations	212		136		117	
R^2^ Nagelkerke	0.057		0.524		0.593	

This table presents Multivariable Cox proportional hazards models assessing predictors of all-cause mortality. Model 1 includes hepatic copper (HCLs); Model 2 adds demographic variables (age, sex, ethnicity, smoking); Model 3 further adjusts for diabetes, dyslipidemia, hypertension, and baseline fibrosis stage. Hazard ratios (HR), 95% confidence intervals (CI), and *p*-values are presented for each covariate. Statistically significant predictors are bolded.

## Data Availability

The original contributions presented in this study are included in the article/Supplementary Material. Further inquiries can be directed to the corresponding author.

## Supplementary Materials

The following supporting information can be downloaded at: https://www.mdpi.com/article/10.3390/nu17182923/s1, Figure S1: Bivariate associations between hepatic copper and fibrosis Measures.
