# Supplementary material for: Hepatic Copper Accumulation Predicts Fibrosis Progression and Mortality in Patients with Metabolic Dysfunction-Associated Steatotic Liver Disease (MASLD)
Source: Nutrients. 2025 Sep 11;17(18):2923. doi: 10.3390/nu17182923 (PMC12472418; doi:10.3390/nu17182923)
Supplement: Supplementary file 1 [file nutrients-17-02923-s001.zip › nutrients-3830502-supplementary.pdf]

Supplementary Figures:

Figures S1a, 1b, 1c, 1d

Figure S1a

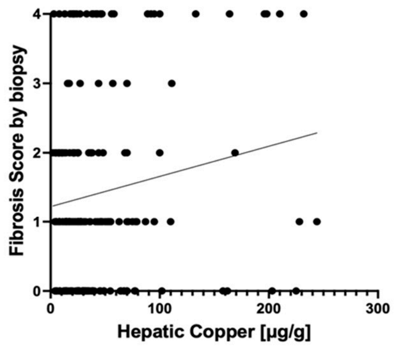

Association is statistically detectable. Slope > 0 (0.004351; 95% CI 0.00052–0.00818; **p = 0.026**), yet **R^2 = 0.023**, meaning X explains only **~2.3%** of the variability in Y.

Figure S1b

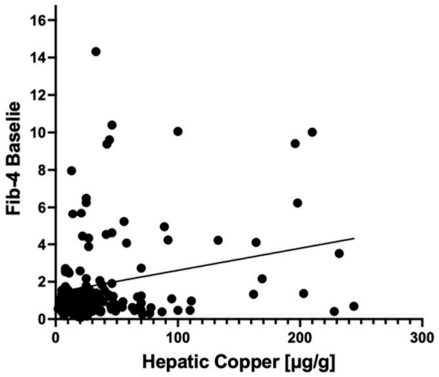

Association is statistically detectable. Slope > 0 (0.0118 (95% CI 0.0035–0.0201), **R^2 = 0.045**, **p = 0.005**

Figure S1c

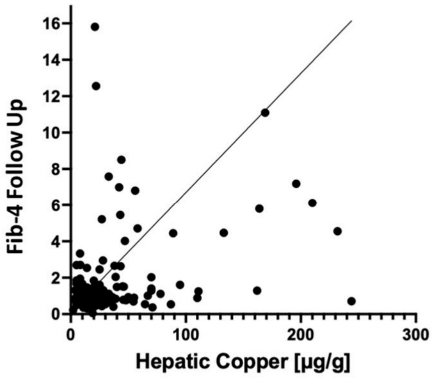

Association is statistically detectable. Slope > 0 (0.0655 (95% CI 0.0376–0.0935), p < 0.0001), **R^2=0.147; p<0.0001**

Figure S1d

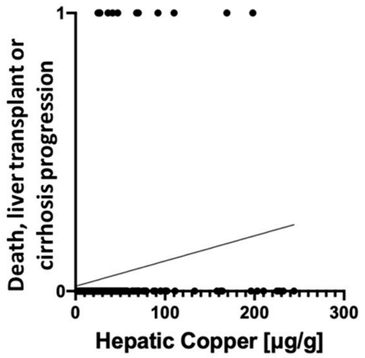

Association is statistically detectable.
Slope > 0 (0.000905 (95% CI 0.000236–0.001574)), **R^2 = 0.032**, **p = 0.0083**

Figure S1: Bivariate associations between hepatic copper and fibrosis Measures.Bivariate associations assessed using ordinary least squares (OLSs) linear regression, fitting $Y = \beta_0 + \beta_1 X + \varepsilon$. The primary effect estimate was the slope (β_1) with 95% confidence intervals (CIs); two-sided tests were used with α = 0.05. Model fit was summarized by R^2 and the residual standard deviation. Assumptions of linearity, homoscedasticity, and approximate normality of residuals were evaluated by residual-vs-fitted and Q-Q plots; influential observations were screened by Cook's distance. For skewed variables, a log-transformation was prespecified as a sensitivity analysis. When multiple pairwise models were fit, false-discovery rate (Benjamini–Hochberg) adjustment was applied. Where variables were ordinal or markedly non-normal, we additionally report Spearman's rank correlation (ϱ) with 95% CI. Analyses were performed in GraphPad Prism (GraphPad Software, San Diego, CA). Accordingly, we tested whether hepatic copper concentration correlates with clinical outcomes and fibrosis measures, fitting prespecified bivariate ordinary least squares (OLS) models of each endpoint on copper. In this cohort, higher hepatic copper is associated with modestly higher fibrosis burden and risk signals, with the clearest relationship at Fib-4 follow-up, but overall effect sizes are small ($R^2 \approx 0.02$–0.15).
